# Gender-Related Barriers and Delays in Accessing Tuberculosis Diagnostic and Treatment Services: A Systematic Review of Qualitative Studies

**DOI:** 10.1155/2014/215059

**Published:** 2014-05-11

**Authors:** Lakshmi Krishnan, Tokunbo Akande, Anita V. Shankar, Katherine N. McIntire, Celine R. Gounder, Amita Gupta, Wei-Teng Yang

**Affiliations:** ^1^Department of Medicine, Johns Hopkins University School of Medicine, Baltimore, MD 21287, USA; ^2^Johns Hopkins Bloomberg School of Public Health, Baltimore, MD 21205, USA; ^3^Center for Clinical Global Health Education, Johns Hopkins University, 600 North Wolfe Street, Phipps 540B, Baltimore, MD 21287, USA

## Abstract

*Background*. Tuberculosis (TB) remains a significant global public health problem with known gender-related (male versus female) disparities. We reviewed the qualitative evidence (written/spoken narrative) for gender-related differences limiting TB service access from symptom onset to treatment initiation. *Methods*. Following a systematic process, we searched 12 electronic databases, included qualitative studies that assessed gender differences in accessing TB diagnostic and treatment services, abstracted data, and assessed study validity. Using a modified “inductive coding” system, we synthesized emergent themes within defined barriers and delays limiting access at the individual and provider/system levels and examined gender-related differences. *Results*. Among 13,448 studies, 28 studies were included. All were conducted in developing countries and assessed individual-level barriers; 11 (39%) assessed provider/system-level barriers, 18 (64%) surveyed persons with suspected or diagnosed TB, and 7 (25%) exclusively surveyed randomly sampled community members or health care workers. Each barrier affected both genders but had gender-variable nature and impact reflecting sociodemographic themes. Women experienced financial and physical dependence, lower general literacy, and household stigma, whereas men faced work-related financial and physical barriers and community-based stigma. *Conclusions*. In developing countries, barriers limiting access to TB care have context-specific gender-related differences that can inform integrated interventions to optimize TB services.

## 1. Introduction

Tuberculosis (TB) comprises a major portion of the global disease burden and is associated with significant gender-related disparities, defined using binary male/female gender categories. In 2011, an estimated 33% of incident TB (2.9 million cases) occurred among women worldwide [1]. While the global case notification rate of TB is lower for women than men, TB is among the top three most significant causes of death among women aged 15 to 44 years [1]. Despite the gender-based disparities in TB statistics and the considerable disease burden among both genders, relatively little attention has been focused on gender-related differences in the epidemiology, diagnosis, treatment, outcomes, or socioeconomic costs of TB [2].

Gender-related disparities in TB and TB care may stem from gender-based differences in the barriers and delays that limit access to TB services. Several reviews suggest that gender differences in barriers that limit access to TB services exist at the individual and provider/system levels. These barriers include the direct and indirect costs of seeking TB services; casual or consistent employment; distance to TB services and access to transportation; stigma surrounding TB diagnosis and its association with HIV; TB-related knowledge and education; distinct gender-specific roles and status in the family; provider degree of suspicion for TB; number and types of providers seen; adherence with national TB program guidelines; and patient satisfaction with TB services [3–11].

Analyses of both qualitative data (primarily based on written or spoken narrative) and quantitative data (primarily based on numbers, frequencies, or mathematical equations) are needed to develop a comprehensive understanding of gender-related differences in the barriers and delays that limit access to TB care. Although previous reviews have found quantitative evidence [3–12], none have systematically reviewed the qualitative evidence. Qualitative data can uniquely inform the nature of quantitative gender-based differences, clarify formative pathways, and may reveal gender-related differences where no quantitative differences were found. Thus, qualitative gender analyses may provide critical insights to inform effective gender-specific interventions.

Our systematic review aims to assess the qualitative evidence for gender-related differences in barriers and delays that limit access to TB diagnostic and treatment services as reported by untreated patients and community members with suspected TB, persons who have presented for TB care, the general population, and health care providers. Using an inductive coding system for qualitative research synthesis, we examined emergent, gender-related themes within defined barriers and delays at the individual and provider/system levels, and our findings have implications for the development and prioritization of integrated gender-specific interventions to optimize the global impact of TB services.

## 2. Methods

### 2.1. Systematic Review Process

#### 2.1.1. Search Strategy

We searched 12 electronic databases for human and English-language articles published between January 1953 and October 2010. Our search strategy was developed for MEDLINE using PubMed with a combination of controlled vocabulary and keyword terms and phrases (see Supplementary Material available online at http://dx.doi.org/10.1155/2014/215059). The strategy was then translated for the Excerpta Medica Database (EMBASE), the Cumulative Index to Nursing and Allied Health Literature (CINAHL), Global Health, Popline, Africa Wide, LILACS, Web of Science, and the inclusive databases of The Cochrane Library using their respective thesaurus terms, synonyms, and keywords. Citations from each database were imported into a reference management system, and duplicates were removed.

#### 2.1.2. Study Selection Criteria

We included qualitative studies that reported on gender-related differences in barriers to and/or delays in accessing TB diagnostic and treatment services and studied human participants aged 15 years or older. We excluded studies that did not provide a gender comparison as well as case reports, editorials, review articles, commentaries, practice guidelines, and studies of treatment compliance and/or outcomes. Participants were defined as persons with diagnosed or suspected TB, persons from either the general population or high-risk populations (e.g., HIV-infected, homeless, and prisoner), or health care providers. Diagnosed TB included both pulmonary and extrapulmonary forms, and TB diagnosis could be made by sputum smear microscopy, culture, or chest X-ray using histopathological or clinical criteria.

#### 2.1.3. Study Selection Process

**Figure 1:**
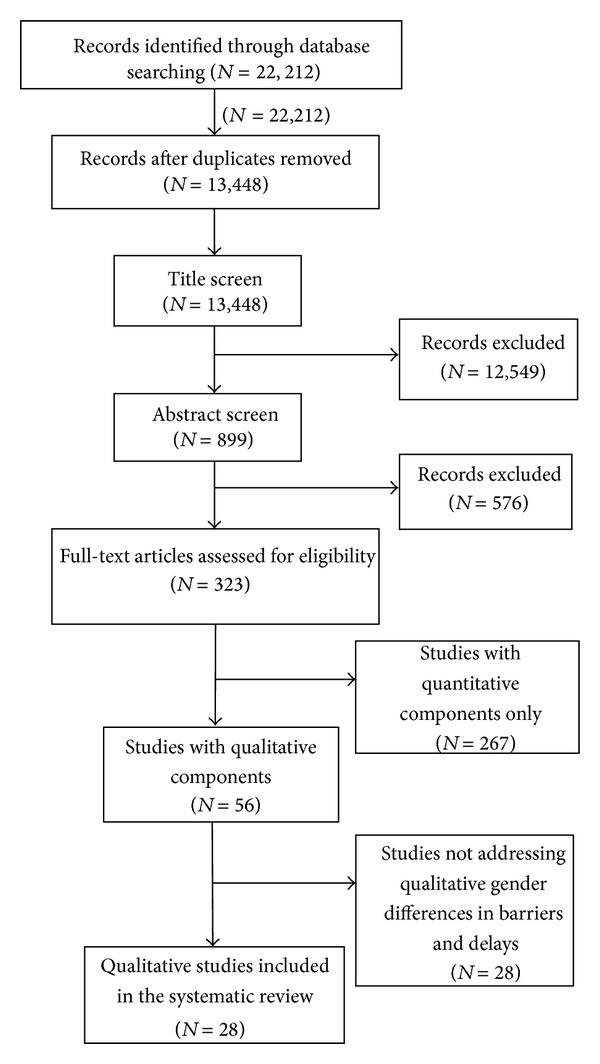
Study selection process.

Following deduplication, studies were screened sequentially by title and abstract and in full-text form (Figure 1). At each stage, two reviewers independently evaluated each study against our study selection criteria. Articles were included or excluded only when both reviewers were in agreement, and conflicts were resolved by a third, independent reviewer (AS). To ensure sufficient concordance between reviewers, a pilot review and reviewer discussion were conducted at each stage before proceeding with the remaining studies. Following the full-text screen, included articles underwent a full-text assessment, which included data abstraction and a study validity assessment.

#### 2.1.4. Data Abstraction and Synthesis

Three reviewers (Lakshmi Krishnan, Tokunbo Akande, and Wei-Teng Yang) independently abstracted data from each included full-text article in duplicate, and any conflicts were resolved through discussion with an additional independent reviewer (AS). Qualitative data were abstracted using a method similar to the “thematic synthesis” approach proposed by Thomas and Harden [13], which includes systematic data extraction involving the translation of concepts between studies, data analysis, and a quality assessment. Study design characteristics were abstracted and summarized, and we used a modified “inductive coding” system for qualitative research synthesis to develop “descriptive” and “analytical” themes within outcomes (our defined barriers and delays). Accordingly, we took findings to be text characterized as “results” or “study findings,” and data were abstracted based on our review questions regarding gender-related differences and barriers to accessing TB diagnostic and treatment services. The general themes from these questions were categorized by outcome type (barrier or delay) and level of impact (individual or provider/system level).

#### 2.1.5. Validity Assessment

We used the validity assessment to examine the quality of studies that inform our review; the assessment was not used to exclude studies. We examined several quality criteria for qualitative research [14–16], and, based on factors determined to critically inform our review process, we developed an adapted version of the Critical Appraisal Skills Programme (CASP) quality assessment tool to assess each study. Our validity assessment tool evaluated research design and purpose, study implementation, and the strength of the evidence used to support the conclusions of the study (Table 1).

### 2.2. Outcomes and Definitions

**Figure 2:**
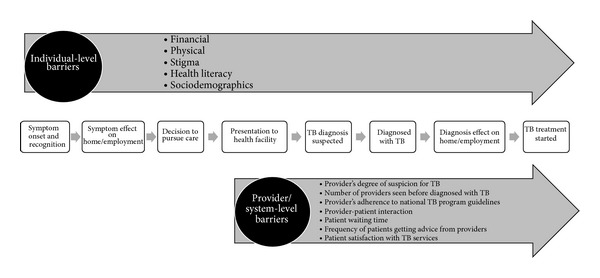
Conceptual framework illustrating barriers and delays that limit access to TB diagnostic and treatment services.  Figure 2 illustrates the conceptual framework of the TB care continuum from symptom onset to treatment initiation that was used to define the barriers and delays that limit access to TB diagnostic and treatment services at the individual and provider/system levels. Individual-level barriers impact access to TB services along the full continuum of TB care, and the provider/system-level barriers impact TB service access from patient presentation to any health care provider through TB treatment initiation. Barriers may contribute to delays between each step along the TB care continuum. Accordingly, we define individual-level delay as the delay between symptom onset and presentation to any health care provider; provider/system delay as the delay between presentation to any health care provider and diagnosis, the delay between presentation to any health care provider and treatment initiation, or the delay between diagnosis and treatment initiation; and combined individual/provider/system delay as the delay between symptom onset and diagnosis or the delay between symptom onset and treatment initiation.

Outcomes were qualitative associations between gender and both barriers and delays that limit access to TB services along the full continuum of TB care from symptom onset through diagnosis and treatment initiation.  Figure 2 presents the conceptual framework that was used to define barriers and delays at the individual and provider/system levels at various time points along the continuum of TB care. Individual-level barriers were defined as follows: financial (the direct or indirect costs of TB care, including costs of travel, diagnosis, and/or treatment as well as the opportunity costs of lost employment, compensation, or household work); physical (distance, travel logistics, and/or access to TB care facilities); stigma (TB-specific sociocultural barriers arising from community or individual prejudice related to TB diagnosis or treatment, including social isolation, marriage prospects, fertility concerns, and association with HIV); health literacy (TB-related knowledge and education); and sociodemographic (age, race, rural versus urban residence, social caste, norms of practice, and social hierarchies). Provider/system-level barriers were defined as any of the following: provider degree of suspicion for TB, number of providers seen before TB diagnosis, provider adherence to national TB program guidelines, provider-patient interaction, patient waiting time, frequency of getting advice, and patient satisfaction with TB services. Delay was defined as any time period between points along the TB care pathway under our conceptual framework, namely, the time period from symptom onset to presentation, diagnosis, or treatment initiation; presentation to diagnosis or treatment initiation; or diagnosis to treatment initiation.

## 3. Results

### 3.1. Study Characteristics

Our search strategy yielded 13,448 citations. Of these, 323 articles were screened in full-text form, and 28 studies [17–44] were included in our review (Figure 1). Overall, the included study characteristics were heterogeneous (Table 2). Studies ranged in size from 22 to 750 participants with women comprising 23–58% of the study population. Regarding study participants, 18 (64%) studies included TB cases or suspects presenting for care, 12 (43%) included community members, and 8 (29%) included health care providers or workers. All studies were published between 1995 and 2010 and were most commonly conducted in the World Health Organization (WHO) Southeast Asian (43%), Western Pacific (32%), and African Regions (25%).

### 3.2. Quality of Qualitative Research Reporting

Based on the validity assessment used to assess the included studies, we found a wide range of quality in methods, experimental design, and result reporting (Table 1). Several studies relied on only one form of data collection, and others based conclusions on a small number of participants (or groups) sampled for the study. In addition, some studies examined normative beliefs related to perceived stigma or barriers based on non-TB patient reports, which may not be representative of the actual barriers experienced by those with TB. There were several issues with qualitative studies including limited sample sizes and conclusions based on insufficient data. The studies that used multiple methods or a mix of qualitative and quantitative methods provided the strongest evidence to inform this review.

### 3.3. Outcomes

All of the included studies reported on individual-level barriers to accessing TB diagnostic and treatment services, and 11 (39%) studies also reported on provider/system-level barriers. Only two studies included data on individual-level delays. Community-based studies tended to report on individual-level barriers, whereas health facilities-based studies provided more provider/system-level evidence. No clear differences emerged between urban and rural environments or between types of health facilities. Emergent gender-related themes were synthesized and summarized by outcome type (barrier or delay) and level of impact (individual level or provider/system level) (Table 3). Key findings are discussed in depth below.

### 3.4. Individual-Level Barriers

Overall, prominent gender-related differences emerged within financial, physical, stigma, and health literacy barriers, and gender-specific sociodemographic themes (i.e., sociocultural norms associated with the status and roles of men and women) were found to directly affect each barrier type (Table 3). Emergent themes are discussed in depth by barrier type with illustrative quotations pertaining to each (Boxes 1, 2, 3, 4, 5, and 6).

#### 3.4.1. Financial

The financial burden of obtaining TB care and pursuing treatment was a widespread theme that affected both genders. Among 10 (36%) studies [17–21, 23, 24, 30, 32, 36], only one [17] found that the direct costs for treatment incurred by women tended to be slightly higher than those incurred by men, suggesting a quantitative systems-based difference in treatment cost by gender. However, there was no gender difference in debt. While most studies described a universal financial cost, a gender differential limited access to TB care. Men tended to experience the financial burden of TB both indirectly and directly because illness limited their ability as breadwinners [18], and seeking TB treatment drained their financial resources [19]. In contrast, women lacked financial independence, which limited access to care. Resource allocation within families was another key concern. As the primary earners, men's health is prioritized. While men and children receive the lion's share of resources, women's lack of financial autonomy and lower status within households deprioritizes their health [20–22], leaving them dependent on spouses, families, or social services.

#### 3.4.2. Physical

Only two (7%) studies explored physical barriers to accessing TB services [20, 23], which are generally linked to financial issues. In both studies, participants reported that the direct and indirect costs of traveling to healthcare facilities (including travel time and inflexible hours of health facilities) affected both men and women. One study [20] reported that men were more heavily affected than women since they are employed outside of their communities and, therefore, experience a greater burden due to travel time, seeking care within their communities, and inflexible clinic hours.

#### 3.4.3. Stigma

Stigma pervasively affects both genders and was reported as a major barrier to accessing TB diagnostic and treatment services. Of 28 studies, 20 (71%) investigated gender-related differences in TB-related stigma [17–20, 22–37]. Among these, 9 (45%) studies found that women experienced greater stigma than men [17, 18, 20, 22, 28, 31–33, 35], 8 (40%) studies found no gender-related difference [19, 24–26, 29, 30, 34, 36], two (10%) studies reported that men faced a greater stigma burden than women [23, 37], and one study reported that women in the community had higher prejudice toward TB patients [27].

Several studies highlighted gender differences in the nature of TB-related stigma. Men reported greater workplace, community, and marital prospect-related stigma, whereas women reported greater stigma with family members as well as psychosocial consequences, feelings of isolation, and lack of proper care from the family [28, 30, 31, 35]. Women were also described as more likely to be stigmatized in communities where the level of family stigma was reported as low, but the community perception of TB as associated with immoral behavior was high [26]. The impact of TB-related stigma on marriage was noted in six studies. Many of these [23, 34, 36, 37] found that TB-related stigma affected the marriage prospects of both genders, with men having a slightly more difficult time finding a wife in areas with a low female/male ratio [23, 37]. Two studies [18, 32] discussed divorce as a direct consequence of TB to be more likely to affect females, and, of these, one study [18] reported that TB-infected females were more likely than TB-infected males to face difficult marital prospects. Another study [27] found that a higher percentage of females had high prejudice towards TB patients compared to males (AOR = 1.4; CI = 1.0–1.9). Notably, TB-related stigma overlapped with health literacy issues. Communal lack of knowledge regarding TB transmission and the concern that TB is incurable or can be passed down to offspring lead to stigma that stymies the marital prospects of all TB patients, regardless of gender.

#### 3.4.4. Health Literacy

Men outstripped women in the domain of TB-related knowledge and education. Of 28 studies, 12 (43%) described gender-related differences in health literacy as barriers to accessing TB services [19, 20, 22, 23, 26, 27, 31–33, 37–39]. Among these, six (50%) found that men had higher levels of TB-related knowledge than women [22, 23, 27, 32, 37, 39]. This finding was largely due to higher male educational attainment and reflects gender-related differences in general literacy. Several studies examined gender-related differences in general education attainment and literacy [20, 31, 37], and two found that men were more educated and/or had higher literacy rates than women [31, 37]. No studies reported that women had higher levels of TB-related knowledge than men. However, one study [20] found that women had more knowledge about accessing health services than men. Two studies found that neither men nor women had much knowledge of TB or its transmission [19, 38].

#### 3.4.5. Sociodemographic

Of 28 studies, 18 (64%) revealed sociocultural factors acting at the individual level. In all studies, the sociocultural context of women's roles and status in society permeated the observed gender differences, clearly demonstrating that norms of practice and social hierarchies lead men and women to face different barriers to accessing TB services [17–20, 22, 23, 28–30, 34–37, 39–43]. Specifically, men exhibited an unwillingness to access health care due to social perceptions described below or inability to leave work. Financial burdens as discussed above also play a role. One study [42] found that cultural beliefs (men believed that the body should be resilient and only sought care when it had succumbed to disease) and the perception that healthcare actions carry few benefits [43] affected male willingness to access treatment. Men were also less likely to access healthcare in situations where their work interfered [34]. For women, the primary barriers included the lack of autonomy, the need to seek permission from family [19, 20, 28], the requirement to be chaperoned to a consultation [40], lack of family support [18, 20, 22, 23, 29, 30, 37, 41], the prioritization of men and children's health [23, 41], and the expectation that an ill wife must take care of her husband, but not vice versa [20, 24]. Notably, when women were able to access healthcare, they often utilized healthcare services more than men because they perceived greater benefits from accessing treatment [43].

### 3.5. Provider/System-Level Barriers

Of 28 studies, 11 (39%) assessed gender-related barriers to accessing TB services at the provider/system level [19, 20, 23, 30, 34, 36, 37, 39–41, 44]. Although these combined barriers were felt by community members and patients to affect women more than men, one study found that all participants agreed that subsidized care played a major role in removing some of these barriers [36], and both genders complained that long waiting times, poor facilities [30], diagnostic unreliability or delay, and deferred or inappropriate treatment [36] hindered health-seeking behavior and treatment initiation. The lack of privacy in health clinics with the use of directly observed therapy (DOTS) posed a particular problem for women who experienced greater embarrassment and anxiety than men and reported this as a barrier to seeking treatment [19, 20]. However, in two studies, both men and women reported that, overall, the treatment at TB centers and government facilities was non-gender-discriminatory and fair [20, 34].

Among studies that surveyed health care providers versus the general community or TB patients, health workers who cared for TB patients believed that system-level barriers such as long waiting times, poor health care facilities, and lack of privacy were the most significant factors preventing their patients from accessing care. In contrast, community members or patients perceived greater individual barriers, especially those centered in the domains of finance and stigma. According to Eastwood et al., health workers felt that women experienced more difficulties than men as a result of such barriers (including cost, travel, and lack of privacy at treatment centers). Onifade et al. corroborated this finding, reporting that healthcare workers overwhelmingly stated that women experienced greater barriers (including stigma) to accessing care than men [20]. One study found that healthcare workers assumed a higher health literacy level among their patients than was actually the case, but this applied to both genders [34].

### 3.6. Individual-Level Delays

Only two studies reported on individual-level delays in TB diagnosis and treatment initiation [36, 44]. One study reported that delays affect both genders [36], and one study reported that men tended to delay communication about a suspected illness, which, in turn, appeared to delay treatment seeking [44].

## 4. Discussion 

Understanding gender-related differences in barriers and delays that limit access to TB care is important to inform interventions designed to optimize the impact of TB services. Our systematic review aimed to assess the qualitative evidence for gender-related differences in the barriers and delays that limit access to TB diagnostic and treatment services at the individual and provider/system levels. Collectively, the 28 studies that informed our review represented developing countries within four WHO regions (AFRO, AMRO, WPRO, and SEARO) and reported on each of our defined individual-level barriers (financial, physical, stigma, health literacy, and sociodemographic), with a subset of 11 studies that also reported on provider/system-level barriers. Our review revealed the nature of gender-related differences found within individual-level barriers, which appear to arise from underlying gender-specific sociodemographic themes. Gender-specific themes were particularly pervasive within financial, stigma, and health literacy barriers, and provider/system-level barriers were also found to disproportionately affect women. Our findings may be used to inform interventions that may be integrated with provider/system-level strategies to improve access to TB services.

Our review revealed that gender-specific barriers to accessing TB diagnostic and treatment services are closely related to sociocultural structures. Approximately two-thirds of the included studies mentioned the imbalance in social norms and power between men and women, and gender-specific sociodemographic themes (i.e., gender-specific social norms, roles, and status) were found to impact all barrier types. In contrast, a systematic review of quantitative studies found that less than 5% of studies addressed sociodemographic barriers to accessing TB services [12]. This disparity between qualitative and quantitative evidence may reflect, in part, the difficulty of quantifying such an intangible concept. Specifically, our review revealed that women experienced barriers due to financial dependence, lack of physical independence, the deprioritization of their health in households, stigma within the household, and relatively low general and TB-related literacy, whereas men primarily experienced community-based stigma and work-related financial and physical barriers, which reflect their higher status/power and role as the primary earner. These findings support the prioritization of the gender empowerment framework as advocated by WHO, UNFPA, and other international organizations, which, in addition to balancing research, aims to create a more balanced power structure between men and women in health care and more broadly [45–47]. Overcoming gender-specific sociocultural barriers is an overarching problem that requires intervention but is a large and challenging task. Thus, understanding that the roles and status of women affect each of the individual-level barrier types, we propose that financial, physical, stigma, and health literacy barriers may be more amenable to direct intervention.

Among the individual-level barriers that limit access to TB services, TB-related stigma was particularly pervasive. The majority of included studies (70%) assessed TB-related stigma, and our review revealed that women faced greater stigma than men when gender-related differences were found. Specifically, men faced more prejudice in the workplace and community, whereas women faced prejudice in the household and experienced more psychosocial consequences of isolation. In addition, stigma was found to impact marriage in gender-specific ways. For men, diminished marriage prospects appeared to be linked to the potential inability to work and support the family, whereas women faced concerns with fertility before marriage and threats of divorce after marriage due to fear of contagion and the inability to perform household chores. Importantly, stigma and health literacy are related, and gender-specific interventions to improve TB-related health literacy may overcome some barriers due to individual-level stigma.

Regarding health literacy, our review revealed a clear gender disparity in TB-related knowledge. Several studies reported that men had a higher level of TB-related health literacy than women, and this disparity corresponded with a gender-related difference in general literacy. This finding has implications for health literacy interventions, which will also impact TB-related stigma. The lack of accurate TB-related knowledge impacts individual and community perceptions of disease and can perpetuate stigma [48]. In addition, both general literacy and health literacy are important predictors of health status [48]. Accordingly, community-level programs that provide education on TB symptomatology, risks, and treatment options will continue to be important. However, to prompt more women to seek TB diagnosis and treatment, existing strategies such as educating trusted traditional healers, using community gatherings as a mode of TB education delivery, and providing early childhood education within schools will need to be supplemented with novel gender-specific approaches. For example, interventions that increase the general literacy of women might expand the uptake and impact of existing TB education campaigns that use written materials to explain TB disease mechanisms and treatment. In addition, to optimize the access and uptake of general literacy and TB education programs among women, tailored health communication strategies need to be developed that take into account the financial dependence, lack of autonomy, deprioritization of care, and household roles that impact women's lives. Integrating health education programs with innovative interventions that foster intrinsic motivation and self-advocacy among women may also help overcome the substantial sociodemographic challenges that women face when seeking TB care.

**Figure 3:**
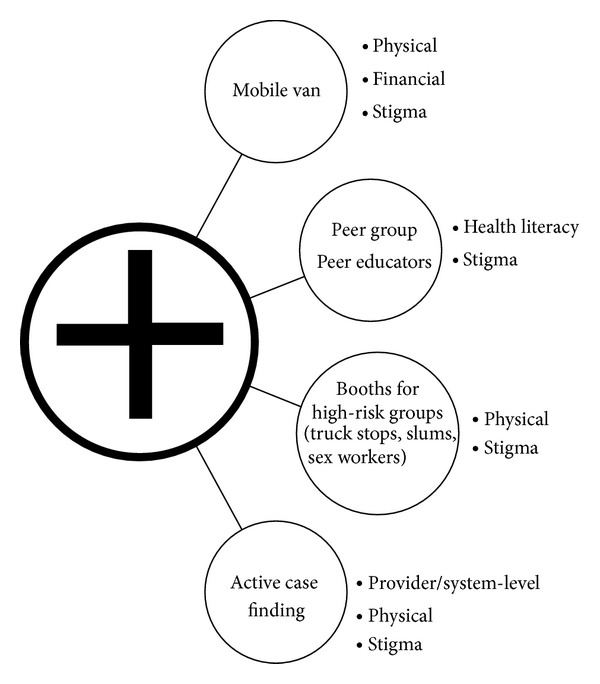
Decentralized model of TB care delivery highlighting suggested interventions to target identified barriers. Figure 3 illustrates the transition from the traditional, centralized model of TB care delivery (central circle with the cross), which uses primary, secondary, and tertiary facility-based TB care (including government hospitals as well as community and nongovernmental organization clinics), to a decentralized model of TB service delivery that incorporates several interventions to overcome the individual-level and provider/system-level barriers identified in this review. Suggested interventions are listed with the specific barriers that each may address.

Gender-specific interventions that address individual-level stigma and health literacy barriers are critical to improve access to TB services. However, overcoming interrelated individual-level financial/physical barriers and provider/system-level barriers must also be prioritized, and a decentralized approach to TB service delivery may be required. Currently, the traditional “top-down” structure delivers TB treatment through government centers, centralized National Government Organization clinics, and community clinics and leaves large gaps in treatment. Our review identified that both genders experience physical barriers due to travel time and inflexible hours of centralized health facilities, which also manifest as gender-specific financial barriers. As the primary household earner, men reported work-related financial barriers, whereas women faced barriers arising from their lower social status, including financial dependence, physical dependence, and the deprioritization of their health care in households. In addition, although fewer studies assessed provider/system-level barriers, our review found that, particularly for women, large central hospitals pose a threat to privacy and may substantiate stigma. Overall, both genders also reported that long wait times, poor facilities, diagnostic delays/unreliability, and deferred/inappropriate treatment hindered access to TB services. Accordingly, we might propose a decentralized TB service delivery structure that integrates provider/system-level treatment options with interventions targeting the gender-specific barriers that limit access to those services (Figure 3). Thus, many financial, physical, stigma, health literacy, and provider/system-level barriers may be overcome by reorienting the way in which TB service delivery interacts with the overarching sociocultural issues that dictate community perceptions of TB.

Strong models of integrated interventions exist and have been designed to address several of our identified individual-level thematic areas as well as the overarching sociocultural context of women's roles and status. Operation ASHA, a program begun in India that is expanding worldwide, addresses financial, physical, stigma, and health literacy barriers and “fills gaps” in treatment by departing from the traditional DOTS model of TB treatment. The program establishes community centers in underserved areas (including slums), provides extensive counseling to destigmatize TB diagnosis and treatment, and leverages trusted community members to assist with education and treatment provision [49]. In Pakistan, programs have engaged the private sector with novel approaches that use laypeople, mobile phone software and incentives, and communication campaigns and have been found to substantially increase TB case notification in urban settings [50]. Overall, these integrated systems of case detection are able to overcome provider/system-level and sociodemographic barriers.

Another possible approach involves leveraging existing HIV programs. In areas where TB-HIV coinfection is prevalent, government and NGO TB treatment programs might adapt strategies used by HIV programs, which, in many communities, have successfully destigmatized the use of government antiretroviral (ARV) treatment centers. Successful strategies include offering HIV counseling at local, community, and public health centers; offering mobile HIV testing and treatment resources in vans or trucks; and providing HIV education in general and neutral community spaces such as schools, community centers, shopping areas, or other gathering places. In addition, one program, the Avahan India AIDS Initiative, has targeted high-risk groups such as male truck drivers and female sex workers by using peer educators and community mobilization to disperse preventive efforts (condoms, education, etc.) and establishing clinical services at gathering points such as truck stops or through a franchised network of private sexually transmitted infection (STI) providers [51]. Tailoring TB intervention efforts to high-risk groups may improve resources allocation and community mobilization, destigmatize TB, and, through the use of peer educators of both genders, may help to neutralize gender-related differences in accessing care.

Further research is urgently needed in the area of gender-related differences in barriers to accessing TB diagnostics and treatment services. The systematic nature of literature identification was the strength of this review but was limited by the number of English-language qualitative studies available in the area of gender-related differences in barriers to TB care. Overall, after a rigorous study selection, we were left with 28 studies from which to extrapolate themes that covered a range of gender-specific issues. Because developed countries were not represented among the included studies, our review may not be globally representative.

However, our review protocol did not exclude developed countries, and the included studies do reflect the regions with the greatest global TB burden. Finally, our study selection criteria excluded studies that lacked a gender comparison. Therefore, studies of pregnant women were largely excluded because only women were involved.

## 5. Conclusions

TB continues to be a significant global public health threat. Moving forward with the post 2015 Millennium Development Goals agenda, the WHO and Stop TB Partnership have proposed a set of interim goals, namely, a 75% reduction of TB mortality and a 40% reduction of TB incidence by 2025 [52]. Improving access to TB care by addressing gender-related barriers will be a key step in this direction. To this end, our systematic review revealed the gender-variable nature and impact of several barriers and their overlapping relationships, thus identifying high-impact targets for interventions that may be tailored to the specific sociocultural context and integrated into decentralized models of TB service delivery. However, to optimize the impact of global TB services, our review also highlights a clear need for further studies. Prospectively designed qualitative gender analyses and standardized methods to study and address gender issues are needed in TB research. In addition, the majority of reviewed studies did not survey HIV-TB coinfected populations. Because both diseases individually cause significant stigma, studies examining the compound effect are critical, including studies to investigate the impact of TB and HIV service programs on barriers and delays in the diagnosis and treatment of both diseases and how TB and HIV service efforts might be coordinated. Lastly, an ongoing focus on gender as a variable affecting access to care must be maintained in both research and intervention, and future work must adopt a broader perspective on gender and address complex gender differences rather than assuming that women alone face greater barriers to treatment than men.

## Supplementary Material

The online Supplementary Material outlines the search strategy used to identify the 13, 448 unique articles that underwent our systematic study selection process. Search terms, limits, and the number of citations identified are provided for each electronic database included in our search strategy.

## Figures and Tables

**Box 1:**
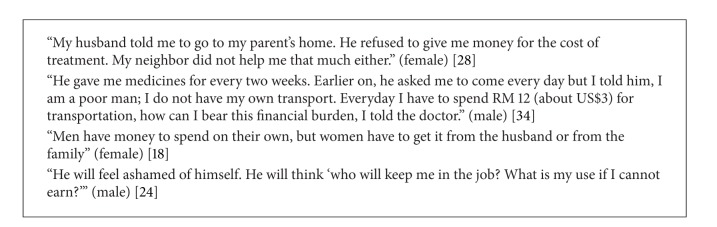
Financial issues.

**Box 2:**
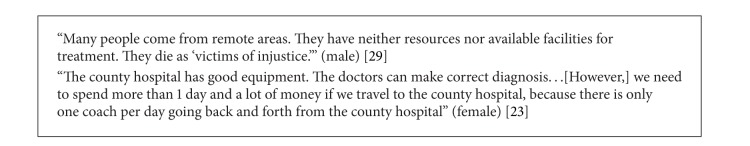
Physical factors.

**Box 3:**
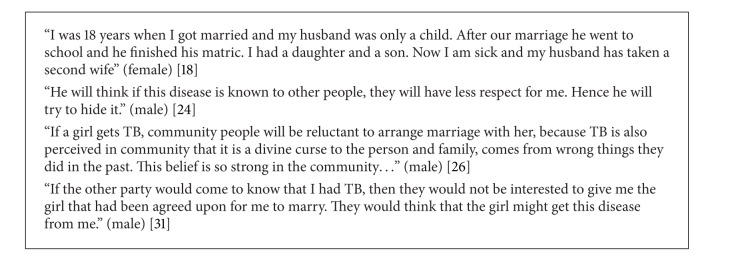
Stigma.

**Box 4:**
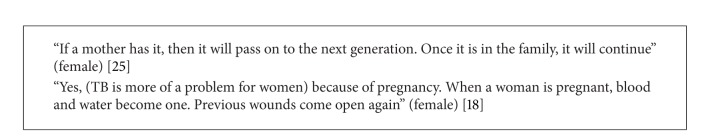
Health literacy.

**Box 5:**
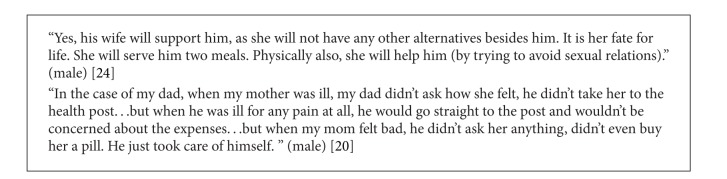
Women's status and roles in society.

**Box 6:**
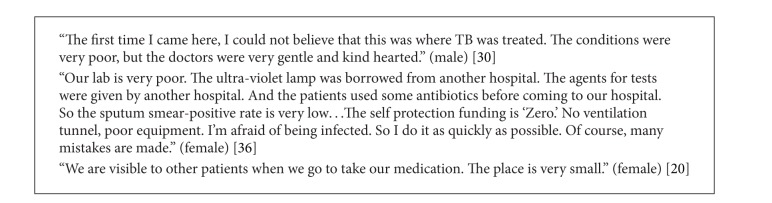
Provider/system-level factors.

**Table 1:** Quality of included studies.

Validity assessment tool^a^		Score		
Item number	Question	Yes (*n*)	No (*n*)	Unclear (*n*)
1	Is there a clear statement of research aims?	28	0	0
2	Is the research design appropriate to address the aims?	21	0	7
3	Are the data collection methods appropriate to obtain the aims?	23	2	3
4	Are the recruitment/sampling strategies appropriate for the aims?	21	0	7
5	Is the study context clearly described?	27	1	0
6	Have ethical considerations been addressed?	17	0	11
7	Is there a clear description of the data collection procedures?	26	2	0
8	Is the data analysis appropriate for research questions?	26	0	2
9	Are the findings clearly presented?	23	5	0
10	Are the claims made supported by sufficient evidence?	24	4	0

^a^After examining several quality criteria for qualitative research [14–16], we developed an adapted 10-question version of the Critical Appraisal Skills Programme (CASP) quality assessment tool based on factors determined to critically inform our review process. Our validity assessment was used to examine the quality of data from the included studies informing this review, not to exclude studies. For questions 1, 4, 5, 6, 7, and 8, studies were scored based on the presence (yes), absence (no), or insufficient information (unclear) regarding the stated criteria. For questions 2, 3, 9, and 10, a subjective review and assessment was performed to determine if the study had any minor or major issues related to compliance with the stated criteria. Accordingly, studies were scored “yes” if either no issues or minor issues were found, “no” if major issues were found, and “unclear” if there was insufficient information.

**Table 2:** Characteristics of included studies.

First author Year	Country WHO region^a^	Number of participants	Percentage of females	Participant characteristics	Research setting	Data collection methods	Type of barrier reported
Atre 2011 [24]	India SEARO	160	50	Community members	Rural	Interviews	Individual-level

Deribew 2010 [27]	Ethiopia AFRO	750	58	Community members	Rural and urban	Questionnaires; FGDs	Individual-level

Mavhu 2010 [42]	Zimbabwe AFRO	40	45	Community members	Urban	Interviews; FGDs	Individual-level

Onifade 2010 [20]	Peru AMRO	53	NR	TB patients; health workers	Urban	Interviews; FGDs	Individual-level Provider-/system-level

Rundi 2010 [34]	Malaysia WPRO	58	53	TB patients; family members; health workers	Rural	Interviews	Individual-level Provider-/system-level

Skordis-Worrall 2010 [44]	South Africa AFRO	8 FGD 6–8 each	NR	TB patients; community members	Urban	FGDs	Individual-level Provider-/system-level

Gosoniu 2008 [28]	Bangladesh, India, Malawi SEARO, AFRO	329	49	TB patients	Rural and urban	Interviews	Individual-level

Jaggarajamma 2008 [29]	India SEARO	276	31	TB patients	Rural and urban	Interviews; FGDs	Individual-level

Somma 2008 [35]	Bangladesh, India, Malawi, Colombia SEARO, AMRO	400	50	TB patients	Rural and urban	Interviews	Individual-level

Weiss 2008 [39]	Bangladesh, India, Malawi SEARO, AFRO	329	49	TB patients	Rural and urban	Interviews	Individual-level Provider-/system-level

Baral 2007 [26]	Nepal SEARO	34	NR	TB patients; family members; community members	Kathmandu Valley	Interviews	Individual-level

Ganapathy 2008 [41]	India SEARO	16 FGD	NR	Community members	Urban	FGDs	Individual-level Provider-/system-level

Karim 2007 [31]	Bangladesh SEARO	102	49	TB patients	Rural	Interviews	Individual-level

Yan 2007 [37]	China WPRO	16–22 FGD 82 interviews	NR	TB patients; community members	Rural	Questionnaires; interviews; FGDs	Individual-level Provider-/system-level

Zhang 2007 [23]	China WPRO	714	50	Rural farmers	Rural	FGDs	Individual-level Provider-/system-level

Fochsen 2006 [40]	India SEARO	22	23	Health care providers	Rural and urban	Interviews	Individual-level Provider-/system-level

Atre 2004 [25]	India SEARO	160	50	Community members	Rural	Interviews	Individual-level

Eastwood 2004 [19]	The Gambia AFRO	45	40	TB patients; health workers	Medical unit	Questionnaires; FGDs	Individual-level Provider-/system-level

Rodríguez-Reimann 2004 [43]	USA AMRO	166	53	Community members w/TB positive family member	Urban	Questionnaires	Individual-level

Sanou 2004 [21]	Burkina Faso AFRO	28 FGD 8–12 each 68 interviews	NR	TB patients; non-TB patients; community members; traditional healers	Rural and urban	Interviews; FGDs	Individual-level

Thorson 2004 [22]	Vietnam WPRO	5 FGD 7-8 each 3 interviews	33	Physicians	General hospital; TB units	Interviews; FGDs	Individual-level

Xu 2004 [36]	China WPRO	16 FGD	31	TB patients; health workers; health providers	Rural	FGDs	Individual-level Provider-/system-level

Long 2001 [32]	Vietnam WPRO	16 FGD 8–10 each	NR	TB patients; non-TB patients	Rural and urban	FGDs	Individual-level

Johansson 2000 [30]	Vietnam WPRO	4 FGD 8–10 each	NR	TB patients; community members	Rural and urban	FGDs	Individual-level Provider-/system-level

Ngamvithayapong 2000 [33]	Thailand WPRO	85	33	Community members; healthcare staff	Rural and urban	FGDs	Individual-level

Long 1999 [38]	Vietnam WPRO	16 FGD 8–10 each	NR	TB patients; non-TB patients	Rural and urban	FGDs	Individual-level

Rajeswari 1999 [17]	India SEARO	304	39	TB patients; non-TB patients	Rural and urban	Interviews; FGDs	Individual-level

Liefooghe 1995 [18]	Pakistan SEARO	48	50	TB patients	Mission hospital	FGDs	Individual-level

FGD: focus group discussion; NR: not reported; TB: tuberculosis; WHO: World Health Organization.

^a^WHO regions: AFRO (African Region), AMRO (Americas Region), SEARO (Southeast Asian Region), and WPRO (Western Pacific Region).

**Table 3:** Summary of emergent gender-related themes by barrier type.

Barrier type	Gender-related themes^a^	
	Gender similarities	Gender differences
*Individual level *		
Financial	Both genders cite finances as a key barrier to seeking care; cost of TB treatment and diagnosis is a shared burden; economic burden affects both genders; cost of healthcare is a gender-wide deterrent to seeking services; and no gender difference in debt is incurred for treatment	Finances have a greater burden on men since they are breadwinners; TB treatment means time away from work and lost earning potential. Less financial independence for women reliant upon families or in-laws; family resource allocation preferring men and children's health above that of women; women's lack of financial autonomy a barrier to accessing care and care decision-making; men with greater access to money and treatment decision-making power; direct treatment costs for women sometimes greater than those for men
Physical	Distance from work and home to treatment facilities was reported as a barrier to accessing care; long traveling times to hospitals is a barrier	Distance from work to treatment affects men more heavily
Stigma	Adverse marital impact of TB-related stigma for both men and women (prospects and spousal support); both genders naming young unmarried people as the group at highest risk of stigma; both genders reporting hiding their diagnosis or describing their disease vaguely for fear of stigma; fear of social isolation reported by both genders; TB stigmatized, but not as much as AIDS; TB stigma not eliminated after treatment	Females expect more stigma in family and reported more isolation, psychosocial consequences, fear of divorce, losing spouses, or compromised marital prospects for unmarried children; TB in women is associated with loose and immoral behavior, leading to greater burden of stigma and more difficulty getting married; women are more likely to hide their diagnosis or delay seeking treatment because of stigma Men expect more stigma at work, sexual relationships, ability to marry
Health literacy	Low education level correlating with greater fear of TB and social isolation; widespread community beliefs that TB is incurable or that TB patients cannot have healthy children; community perceptions that even treated TB can harm offspring, leading to limited marriage prospects	Higher proportion of females displaying prejudice towards TB due to limited knowledge; women and the young with less knowledge than men and the elderly; men with greater formal education and TB knowledge than young and older women; women more likely to regard TB as fatal or incurable; women with limited knowledge in health seeking; men knowing more about HIV/TB transmission than women
Sociodemographic barriers	None	Women need to ask permission from husbands or elders to seek treatment; treatment of children and men is prioritized; diagnosed women receive less family support than men; women are expected to care for husbands with TB, whereas men are not expected to care for wives with TB; more males report that family members have a positive attitude towards their disease; men in societies where masculine resilience is valued are more likely to delay seeking treatment
Provider/system level	Both genders report long waiting times and poor conditions of TB facilities, unreliability of TB diagnostics as barriers; several studies reported government facilities as gender-neutral and fair	Women are more affected by lack of privacy in health facilities; women are more likely to perceive female health care workers as sympathetic and adhere to treatment; DOTS is more distressing for women; women are more likely to consult traditional healers, self-medicate, or use private physicians over government facilities

AIDS: autoimmune deficiency syndrome; DOTS: directly observed therapy; HIV: human immunodeficiency virus; TB: tuberculosis.

^a^Several independent reviewers identified qualitative gender-related themes using “inductive coding” and extracted specific barriers (i.e., individual-level financial, stigma, physical, health literacy, sociodemographic, and provider/system-level barriers). Emergent themes were compiled and then synthesized in Table 3.
